# Intratumoral and peritumoral radiomics analysis for preoperative Lauren classification in gastric cancer

**DOI:** 10.1186/s40644-020-00358-3

**Published:** 2020-11-23

**Authors:** Xiao-Xiao Wang, Yi Ding, Si-Wen Wang, Di Dong, Hai-Lin Li, Jian Chen, Hui Hu, Chao Lu, Jie Tian, Xiu-Hong Shan

**Affiliations:** 1 Department of Radiology, Affiliated People’s Hospital of Jiangsu University, Zhenjiang, China; 2 CAS Key Laboratory of Molecular Imaging, Beijing Key Laboratory of Molecular Imaging, The State Key Laboratory of Management and Control for Complex Systems, Institute of Automation, Chinese Academy of Sciences, Beijing, China; 3 School of Artificial Intelligence, University of Chinese Academy of Sciences, Beijing, China; 4 Zhuhai Precision Medical Center, Zhuhai People’s Hospital (affiliated with Jinan University), Zhuhai, China; 5 Beijing Advanced Innovation Center for Big Data-Based Precision Medicine, School of Medicine and Engineering, Beihang University, Beijing, China; 6 Department of Medical Imaging, Medical College of Jiangsu University, Zhenjiang, China; 7 Engineering Research Center of Molecular and Neuro Imaging of Ministry of Education, School of Life Science and Technology, Xidian University, Xi’an, China

**Keywords:** Lauren classification, Radiomics, Peritumoral analysis, Gastric cancer, Computed tomography

## Abstract

**Background:**

Preoperative prediction of the Lauren classification in gastric cancer (GC) is very important to the choice of therapy, the evaluation of prognosis, and the improvement of quality of life. However, there is not yet radiomics analysis concerning the prediction of Lauren classification straightly. In this study, a radiomic nomogram was developed to preoperatively differentiate Lauren diffuse type from intestinal type in GC.

**Methods:**

A total of 539 GC patients were enrolled in this study and later randomly allocated to two cohorts at a 7:3 ratio for training and validation. Two sets of radiomic features were derived from tumor regions and peritumor regions on venous phase computed tomography (CT) images, respectively. With the least absolute shrinkage and selection operator logistic regression, a combined radiomic signature was constructed. Also, a tumor-based model and a peripheral ring-based model were built for comparison. Afterwards, a radiomic nomogram integrating the combined radiomic signature and clinical characteristics was developed. All the models were evaluated regarding classification ability and clinical usefulness.

**Results:**

The combined radiomic signature achieved an area under receiver operating characteristic curve (AUC) of 0.715 (95% confidence interval [CI], 0.663–0.767) in the training cohort and 0.714 (95% CI, 0.636–0.792) in the validation cohort. The radiomic nomogram incorporating the combined radiomic signature, age, CT T stage, and CT N stage outperformed the other models with a training AUC of 0.745 (95% CI, 0.696–0.795) and a validation AUC of 0.758 (95% CI, 0.685–0.831). The significantly improved sensitivity of radiomic nomogram (0.765 and 0.793) indicated better identification of diffuse type GC patients. Further, calibration curves and decision curves demonstrated its great model fitness and clinical usefulness.

**Conclusions:**

The radiomic nomogram involving the combined radiomic signature and clinical characteristics holds potential in differentiating Lauren diffuse type from intestinal type for reasonable clinical treatment strategy.

**Supplementary Information:**

The online version contains supplementary material available at 10.1186/s40644-020-00358-3.

## Background

Gastric cancer (GC) is one of the most common cancers and its mortality rate ranks third in the GLOBOCAN 2018 database [1]. The Lauren classification system is one of the simple and effective methods for clinical histopathological classification of GC [2]. Generally, the Lauren classification is divided into intestinal type, diffuse type, and mixed type according to the histological morphology and cell characteristics of GC [2]. Research has reported that the prognosis of GC is better for intestinal type compared with diffuse type, especially in early stage, and diffuse type GC exhibits a higher recurrence rate than intestinal type [3].

It’s worth noting that the histological changes of intestinal and diffuse type GC are not only significantly different in the tumor area, but also in the peritumoral gastric mucosa. Compared with diffuse type, gastric mucosa adjacent to the intestinal type has very few normal or near-normal structures. The mucosa around diffuse type GC often displays high fold formation, which is covered by gastric surface epithelium. However, similar fold formation can only be encountered exceptionally in intestinal type. On the other hand, the intestinal metaplasia of intestinal type in the surrounding mucosa is more frequent and widespread than that of diffuse type [2].

If the intestinal type or diffuse type is known before operation, it will be of great significance to the choice of therapy, the evaluation of prognosis, and the improvement of quality of life. However, the Lauren classification cannot be accurately diagnosed until after biopsy or surgery. Therefore, preoperative prediction of the Lauren classification is particularly important but challenging.

In dynamic contrast-enhanced magnetic resonance imaging (DCE-MRI) of GC, extracellular and extravascular volume fraction (Ve) were calculated from the tumor region of interest (ROI), and the diffuse type showed a higher Ve than the intestinal type [4]. However, the DCE-MRI parameters of GC were measured in the ROIs on one selected axial section of the perfusion maps, which may not completely represent the entire tumor. Liu et al.’s study [5] on CT-based texture analysis extracted features from the tumor on arterial phase and portal venous phase CT image slices that depicted the largest area of the lesion. Their results showed that entropy and standard deviation in arterial phase and maximum attenuation, mean attenuation, all percentiles, and mode in portal venous phase, could serve as noninvasive and promising characteristics in the Lauren classification prediction. However, the circulatory state of each patient was different, thus there were many differences between arterial and venous phases measured with uniform delay time, which makes it difficult for application. Also, the study did not incorporate peritumoral features, leading to an incomprehensive research design.

Radiomics utilizes automated quantitative characterization algorithms to transform a large number of excavatable spatial ROI-based image data into representative and effective radiomic features [6]. Recent advancements in radiomics have provided new ideas for individualized management of GC, including lymphatic metastasis prediction [7, 8], distant metastasis prediction [9], therapeutic response evaluation [10], and prognostic evaluation [11, 12]. These studies highlighted the value of radiomics, suggesting that radiomics could be a potential tool for the Lauren classification in GC.

From the above, there have been no researches that predicted the Lauren classification in GC based on intratumoral and peritumoral CT radiomics so far. In the current study, we developed and validated a radiomic nomogram combining intratumoral, peritumoral, and partial clinical information for noninvasive prediction of the Lauren classification in GC patients preoperatively.

## Methods

### Enrollment of patients

This retrospective study was ethically granted by the review board and the informed consents were waived. Patients were enrolled according to the inclusion and exclusion criteria below. The inclusion criteria: (1) pathologically confirmed GC patients who had undergone surgery; (2) patients who had received standard abdominal CT examination within 1 week before surgery. The exclusion criteria: (1) patients who had received preoperative chemotherapy, radiotherapy, chemoradiotherapy or other treatments; (2) patients concurrent with other types of abdominal tumors; (3) patients with incomplete clinical information; (4) patients with low tension drug contraindication, such as glaucoma, anterior glandular hypertrophy, etc.; (5) patients with allergic history of iodine contrast agent; (6) Lauren mixed type patients. A patient recruitment diagram is depicted in Supplemental Figure 1 (see Additional file 1).

Total 539 GC patients (389 males and 150 females; mean age, 63.5 years) were consecutively enrolled between December 2011 and December 2017. CT T stage (CT_T_) and CT N stage (CT_N_) were defined according to the Union for International Cancer Control (UICC) / American Joint Committee on Cancer (AJCC, 5th edition) guidelines. The whole excised specimen after surgery was stained with hematoxylin-eosin (HE) and examined by the pathologists to determine the Lauren classification.

In this study, all the 539 patients were randomly allocated into a training cohort of 377 cases and a validation cohort of 162 cases at a 7:3 ratio. Lauren diffuse type was marked with label ‘1’, and intestinal type was labeled as ‘0’ in machine learning procedures.

### Tumor segmentation on CT images

Detailed CT protocols and acquisition procedure are described in Supplemental Method 1 (see Additional file 1). Manual segmentation was performed on the tumor on venous phase transverse CT images using ITK-SNAP software (version 3.6, http://www.itksnap.org). For the tumor ROIs, radiologists (Y.D. and J.C.) reviewed all the CT image slices of a patient and selected one slice with the largest tumor area to segment under the supervision of a senior radiologist (X.H.S.). The ROI should cover the whole area of the tumor. Y.D. subsequently delineated the tumor ROIs for all the 539 patients and repeated the segmentation procedure after 1 month on 30 randomly selected patients. J.C. performed the segmentation on the same 30 cases afterwards. The segmentations for all the patients along with the randomly selected 30 cases were conducted following the above segmentation methodology and finally approved by X.H.S. They were all blind to other information of patients when they determined the tumor ROIs.

**Fig. 1:**
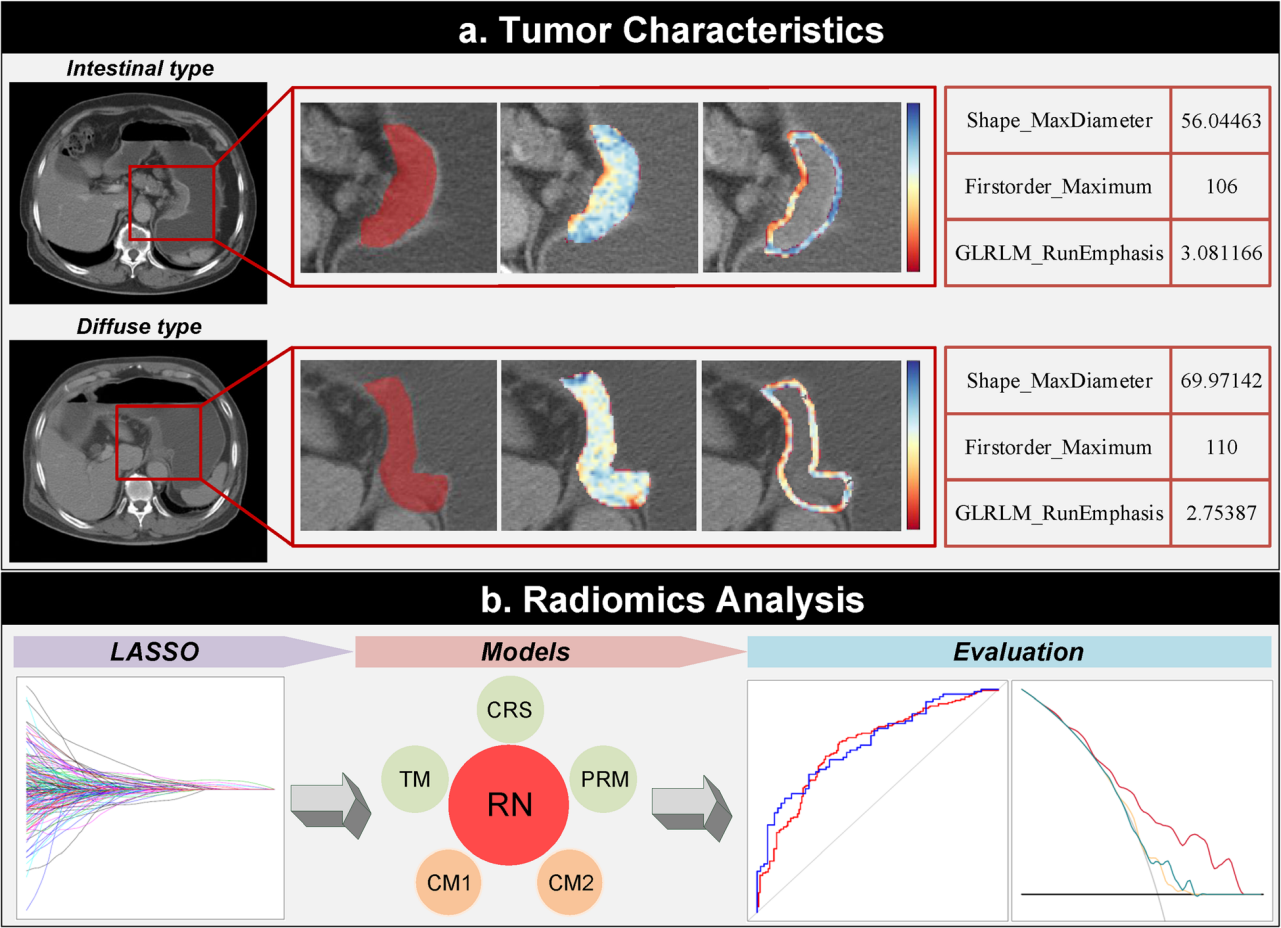
Experimental design flowchart. LASSO, least absolute shrinkage and selection operator; RN, radiomic nomogram; CM1, clinical model 1; CM2, clinical model 2; CRS, combined radiomic signature; TM, tumor-based model; PRM, peripheral ring-based model

Considering the histopathological changes in the immediate vicinity of tumors [13–15], a peripheral ring was automatically created with dilatation and shrinkage by 2 pixels on the previously defined tumor boundary for each patient [16], resulting in a peritumoral ring of 4 pixels (Fig. 1a). We also created peripheral rings for the above selected 30 images segmented by the two radiologists for the subsequent reliability and reproducibility tests.

### Intratumoral and peritumoral radiomic feature extraction

All the CT images were downsampled to a pixel spacing of 3.0 × 3.0 × 3.0 mm^3^ with B-spline interpolation algorithm to ensure an isotropic voxel spacing for the reproducibility of feature extraction [17]. The pixel values were discretized into equally spaced bins using a fixed bin width of 25 Hounsfield Units to eliminate the influence of different grayscale ranges and ensure better comparability. In view of the single layer sketch, two sets of two-dimensional (2D) radiomic features were extracted from original tumor ROIs and derived peripheral rings for all the patients, respectively. Final radiomic features were mainly composed of three groups: shape features, first-order features, and texture features. Detailed information for radiomic feature extraction filters is summarized in Supplemental Method 2 (see Additional file 1). Algorithms were provided in PyRadiomics (version 2.1.1) and implemented in Python 3.6 (https://www.python.org). The radiomics analysis workflow is shown in Fig. 1.

Radiomic feature extraction was also done for tumor ROIs and peripheral rings from the 30 cases segmented by two radiologists, respectively. Intraclass correlation coefficients (ICCs) based on a multiple-rating, consistency, 2-way random-effects model were calculated to assess the stability and reproducibility of radiomic features. For both tumor ROIs and peripheral rings, we only reserved radiomic features with ICC estimates > 0.75, considering their good reliability.

### Feature selection and radiomic signature construction

Numerical radiomic features were first standardized by z-score method using the mean and standard deviation parameters calculated from the patients in the training cohort. Feature selection and radiomic signature construction were conducted in the training cohort.

For simplification and generalization, we only adopted the least absolute shrinkage and selection operator (LASSO) method with 10-fold cross-validation, which is commonly used in feature selection. Herein, the two sets of radiomic features from tumor ROIs and peripheral rings were put together for feature selection. Then, key features screened out were fed to a multivariate logistic regression, giving a final linear formula via adding up corresponding features weighted by respective regression coefficients to calculate a combined radiomic signature per patient.

Meanwhile, a tumor-based model and a peripheral ring-based model were established by the features selected from the tumor ROIs and peripheral rings following the same methodology as the combined radiomic signature (LASSO and logistic regression), respectively.

### Development of individualized radiomic nomogram

Clinical characteristics for the 539 patients are summarized in Table 1, all of which were taken into consideration for building a more efficient radiomic nomogram. Significant clinical factors were highlighted by univariate analysis in the training cohort. Then the combined radiomic signature and selected clinical factors were merged by a multivariate logistic regression, giving the potential calculation formula for radiomic nomogram.

**Table 1:** Clinical characteristics of gastric cancer patients in the training and validation cohorts

Clinical characteristics	Training cohort (***n*** = 377)		***p*** value	Validation cohort (***n*** = 162)		***p*** value
	Intestinal type (*n* = 173)	Diffuse type (*n* = 204)		Intestinal type (*n* = 70)	Diffuse type (*n* = 92)	
**Age, mean ± SD, years**	65.2 ± 8.8	62.2 ± 9.6	0.0042^*^	64.3 ± 8.8	62.6 ± 11.0	0.3045
**Sex, No. (%)**			0.0702			0.8752
**male**	130 (75.1)	135 (66.2)		54 (77.1)	70 (76.1)	
**female**	43 (24.9)	69 (33.8)		16 (22.9)	22 (23.9)	
**CT T stage, No. (%)**			0.0247^*^			0.6810
**T1–2**	53 (30.6)	38 (18.6)		18 (25.7)	19 (20.7)	
**T3**	75 (43.4)	102 (50.0)		25 (35.7)	38 (41.3)	
**T4**	45 (26.0)	64 (31.4)		27 (38.6)	35 (38.0)	
**CT N stage, No. (%)**			0.0061^*^			0.0430^*^
**N0**	46 (26.6)	45 (22.1)		10 (14.3)	22 (23.9)	
**N1**	80 (46.2)	72 (35.3)		35 (50.0)	26 (28.3)	
**N2**	40 (23.1)	63 (30.9)		20 (28.6)	34 (37.0)	
**N3**	7 (4.0)	24 (11.8)		5 (7.1)	10 (10.9)	

NOTE. *p* values are derived from univariate analysis between each clinical characteristic and the Lauren classification. *Abbreviations*: *SD* Standard deviation, *CT* Computed tomography. *p value < .05

For comparison, clinical model 1 was built by CT_T_ and CT_N_, both of which are significant predictors in GC. Clinical model 2 was built by the selected univariately significant clinical factors, thus the incremental value of the combined radiomic signature to radiomic nomogram could be verified.

### Performance evaluation and comparison of different predictive models

Performances of the 6 predictive models (radiomic nomogram, combined radiomic signature, tumor-based model, peripheral ring-based model, clinical model 1, and clinical model 2) were evaluated and compared in terms of discrimination ability and clinical usefulness. To assess the discrimination ability, receiver operating characteristic (ROC) curves as well as corresponding area under ROC curves (AUC) were given for both cohorts. Accuracy, specificity, and sensitivity results were also attached. We adopted Delong-test to compare the predictive performance between each two models. To verify the good fitness of model predictive outputs with actual values, calibration curves were conducted for the radiomic nomogram. To quantify the usefulness in clinical trials, decision curves were conducted in the validation cohort by calculating the net benefits at some threshold probabilities [18, 19].

We further validated the promotion in performance of radiomic nomogram brought by the combined radiomic signature by integrated discrimination improvement (IDI) [20], an effective measurement for performance improvement by adding markers to some existing predictors.

### Statistical analysis

In univariate analysis, Mann-Whitney U test was adopted for continuous clinical factor (age), and Chi-squared test or Fisher exact test were applied for categorical variables (sex, CT_T_, and CT_N_). Multiple comparison corrections were done to adjust univariate *p* values by Bonferroni correction and Benjamini & Hochberg (BH) correction with 4 tests, respectively. A two-tailed *p* value < 0.05 represented a statistical significance. All statistical analysis was performed on R software (version 3.4.3; https://www.r-project.org) and R packages used in our work are summarized in Supplemental Method 3 (see Additional file 1).

## Results

### Clinical characteristics

Baseline clinical characteristics for patients in both the training and validation cohorts are summarized in Table 1. Patients with Lauren intestinal type covered 45.9% (173/377) and 43.2% (70/162) of the training and validation cohort, respectively. And patients with diffuse type occupied 54.1% (204/377) and 56.8% (92/162), respectively. No significant difference (*p* = 0.5666, Chi-square test) in the Lauren classification was captured between the two cohorts. Also, the training and validation cohort were balanced in age (*p* = 0.9868), sex (*p* = 0.1376), CT_T_ (*p* = 0.0879), and CT_N_ (*p* = 0.4390). In the univariate analysis based on the training cohort, the Lauren classification had significant associations with age (*p* = 0.0042), CT_T_ (*p* = 0.0247), and CT_N_ (*p* = 0.0061) while sex (*p* = 0.0702) was excluded. The adjusted *p* values by multiple comparison corrections were given in Supplemental Table 1 (see Additional file 1).

### Feature discovery and radiomic signature building

A total of 2074 (1037 + 1037) radiomic features were initially extracted from tumor ROIs and peripheral rings on each CT image, respectively, including 28 (14 + 14) shape features, 396 (198 + 198) first-order features, and 1650 (825 + 825) texture features. Data cleaning was done to remove radiomic features of all zeros or all the same values. Then with an ICC > 0.75 as a reliability standard, 232 tumor-based features and 206 peripheral ring-based features were included for the subsequent feature selection. LASSO method identified 3 potential features, including 2 first-order features from tumor ROIs and 1 shape feature from peripheral rings. Detailed descriptions for the 3 features are given in Supplemental Method 4 (see Additional file 1) and the formulas for the combined radiomic signature, tumor-based model, and peripheral ring-based model are given in Supplemental Method 5 (see Additional file 1).

### Development of individualized radiomic nomogram

**Fig. 2:**
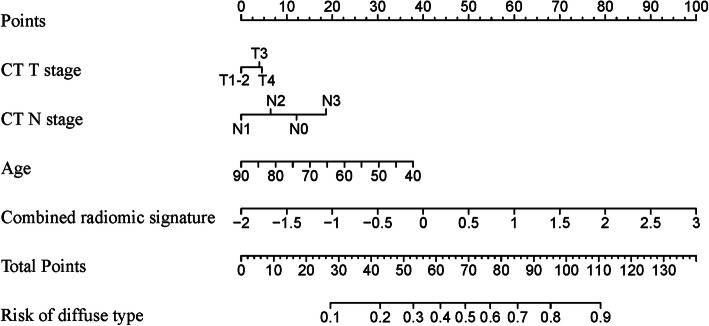
Visualization of the radiomic nomogram, indicating that gastric cancer patients were tended to be predicted as Lauren diffuse type with younger age, advanced CT T stage, and advanced CT N stage. CT, computed tomography

Three clinical factors identified by univariate analysis (age, CT_T_, and CT_N_) along with the combined radiomic signature constructed the radiomic nomogram. The formula for radiomic nomogram is shown in Supplemental Method 5 as well (see Additional file 1). Further, the radiomic nomogram is visualized in Fig. 2. For comparison, clinical model 1 was built by CT_T_ and CT_N_. Clinical model 2 was established by age, CT_T_, and CT_N_.

### Performance evaluation and comparison of different predictive models

**Fig. 3:**
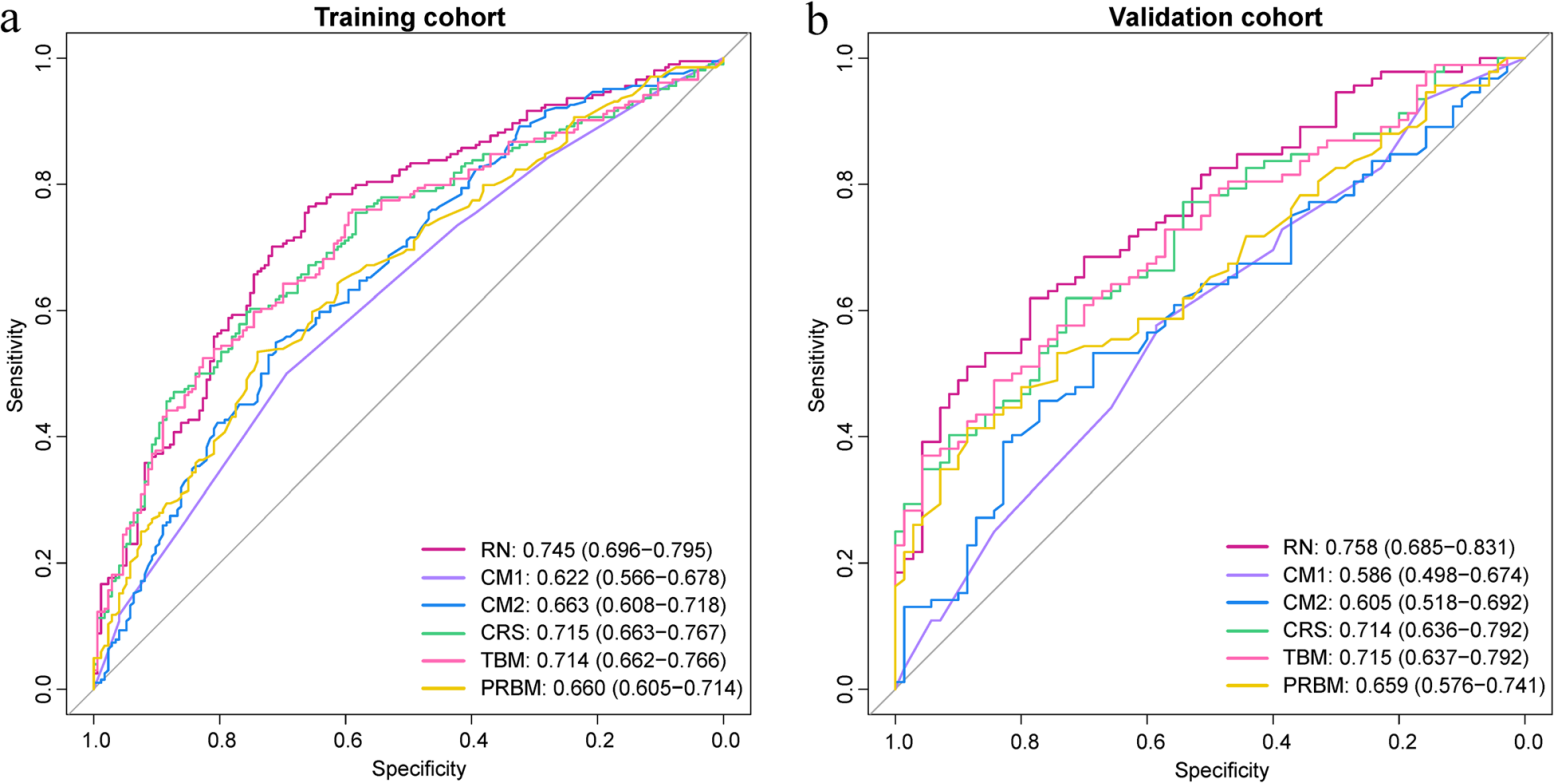
Receiver operating characteristic curves of 6 predictive models in the **a** training and **b** validation cohort. RN, radiomic nomogram; CM1, clinical model 1; CM2, clinical model 2; CRS, combined radiomic signature; TM, tumor-based model; PRM, peripheral ring-based model

As shown in Table 2 and Fig. 3, the combined radiomic signature achieved a fine discriminative performance with an AUC of 0.715 (95% confidence interval [CI], 0.663–0.767) in the training cohort and 0.714 (95% CI, 0.636–0.792) in the validation cohort. To further validate the representative performance of the combined radiomic signature, we re-conducted feature selections and radiomic signature constructions based on 10-time dataset random allocations, respectively. The results were given in Supplemental Table 2 (see Additional file 1). The final radiomic nomogram surpassed all the other models with a training AUC of 0.745 (95% CI, 0.696–0.795) and a validation AUC of 0.758 (95% CI, 0.685–0.831). Also, the radiomic nomogram indeed possessed the highest predictive accuracy. What’s worth noting is that the radiomic nomogram boasted a significantly improved sensitivity (0.765 for training cohort and 0.793 for validation cohort), which represented that we could better identify GC patients with Lauren diffuse type.

**Table 2:** Performance of different predictive models

Models	Training cohort				Validation cohort			
	AUC (95% CI)	ACC	SPE	SEN	AUC (95% CI)	ACC	SPE	SEN
Radiomic nomogram	0.745 (0.696–0.795)	0.716	0.659	0.765	0.758 (0.685–0.831)	0.673	0.514	0.793
Combined radiomic signature	0.715 (0.663–0.767)	0.671	0.757	0.598	0.714 (0.636–0.792)	0.642	0.743	0.565
Tumor-based model	0.714 (0.662–0.766)	0.663	0.827	0.525	0.715 (0.637–0.792)	0.630	0.800	0.500
Peripheral ring-based model	0.660 (0.605–0.714)	0.629	0.740	0.534	0.659 (0.576–0.741)	0.617	0.729	0.533
Clinical model 1 (CT_T_ + CT_N_)	0.622 (0.566–0.678)	0.589	0.694	0.500	0.586 (0.498–0.674)	0.574	0.586	0.565
Clinical model 2 (age + CT_T_ + CT_N_)	0.663 (0.608–0.718)	0.623	0.711	0.549	0.605 (0.518–0.692)	0.574	0.586	0.565

NOTE. *Abbreviations*: *AUC* Area under the curve, *CI* Confidence interval, *ACC* Accuracy, *SPE* Specificity, *SEN* Sensitivity, *CT*_*T*_ CT T stage, *CT*_*N*_ CT N stage

**Fig. 4:**
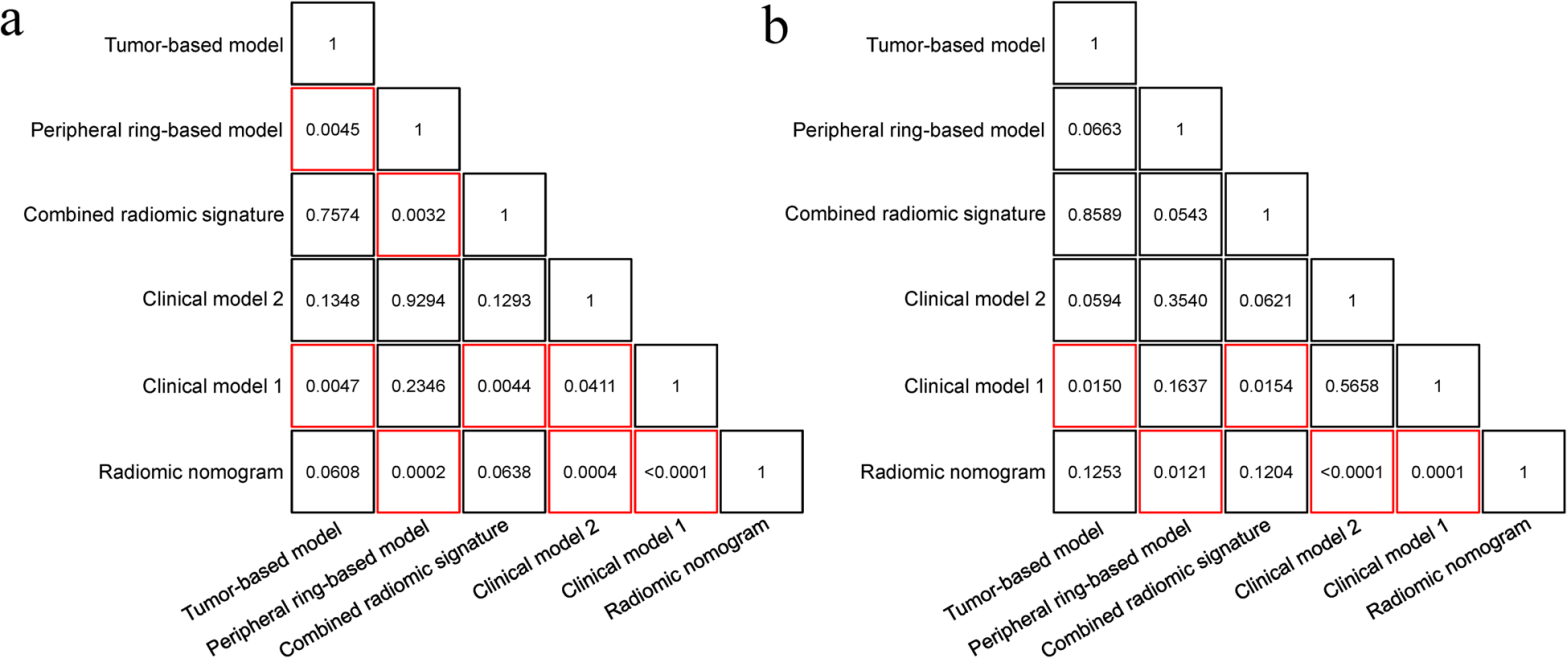
Delong-test results between each two models in **a** training and **b** validation cohort. Red boxes represent *p* values < 0.05

Delong-test results in Fig. 4a and b indicated that there were significant differences between predictive performance of radiomic nomogram and that of clinical model 1, clinical model 2, and peripheral ring-based model in both cohorts. The combined radiomic signature achieved equivalent AUCs compared to the tumor-based model, yet demonstrating promotions in both accuracy and sensitivity results. Meanwhile, the combined radiomic signature performed better than the peripheral ring-based model as indicated by AUCs, though this was confirmed insignificant in the validation cohort (*p* = 0.0543).

As to the association between radiomic nomogram and the combined radiomic signature, an IDI of 5.71% in the validation cohort demonstrated the improvement from the combined radiomic signature to radiomic nomogram though Delong-test showed no significant difference (Fig. 4b, *p* = 0.1204). Besides, the promotion in performance of radiomic nomogram brought by the combined radiomic signature was again verified by an IDI of 17.73% along with the Delong-test *p* value of < 0.0001 in the validation cohort.

**Fig. 5:**
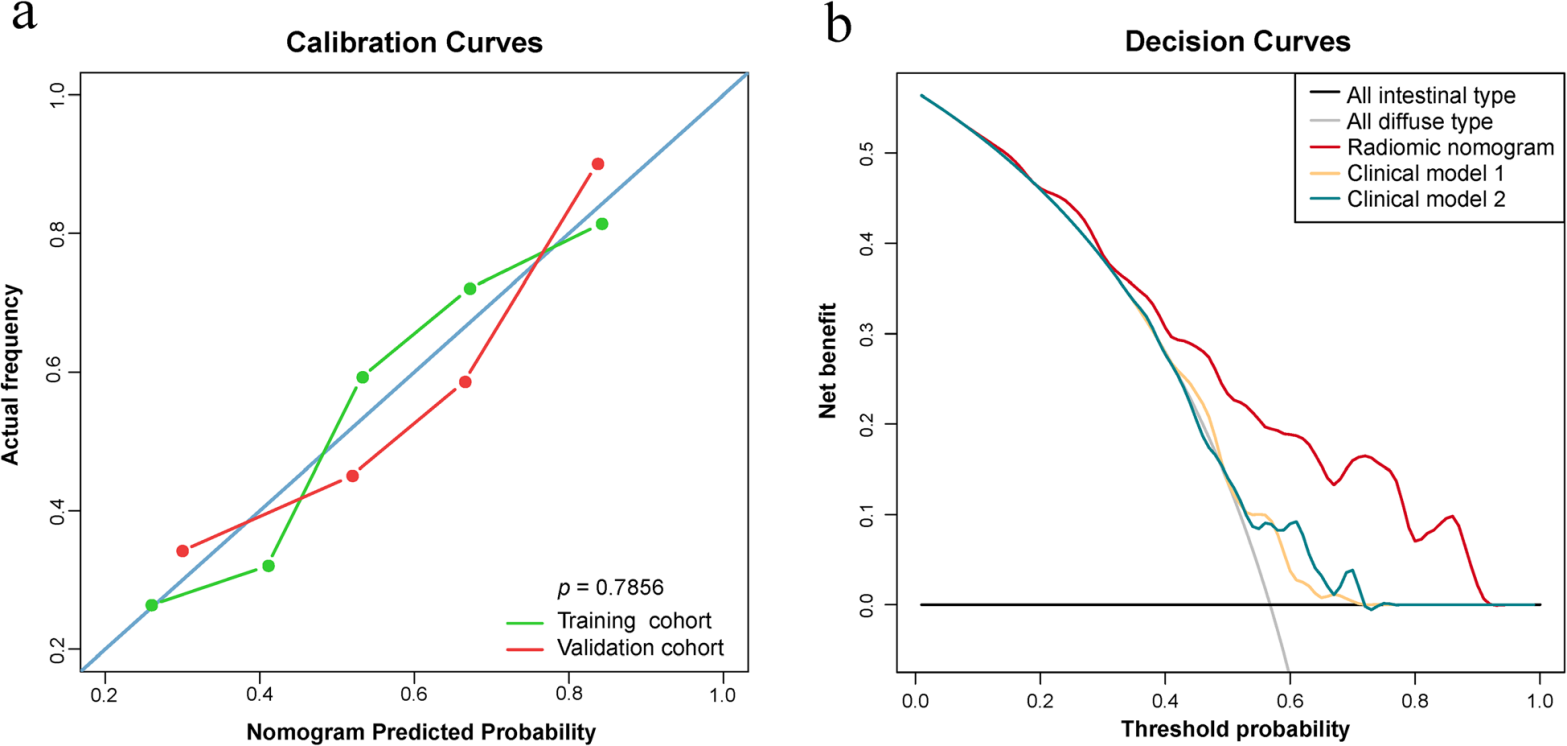
**a** Calibration curves for radiomic nomogram in both cohorts. **b** Decision curve analysis for radiomic nomogram and clinical models in the validation cohort

Calibration curves for the radiomic nomogram showed good fitness (Fig. 5a). Decision curve analysis (Fig. 5b) in the validation cohort indicated that if the threshold probability of a patient was within the whole range of 0.0–1.0, using the radiomic nomogram to predict the Lauren classification added more net benefit to make the decision of whether to undergo treatment than the two clinical models.

## Discussion

In this study, we established a radiomic nomogram to identify the Lauren classification in GC preoperatively. The radiomic nomogram is an easy-to-use tool for individualized decision making, which shows a very important guidance value for the formulation of treatment plans.

For many years, the identification of Lauren types in GC mainly relies on the pathological diagnosis by clinical oncologists, which usually requires pathological biopsy that harms the body. Although the pathological results of gastroscopic biopsy were also obtained before operation, few tissue specimens were obtained, and the consistent rate of the Lauren classification was only 64.7% between biopsy and surgical samples [21]. In recent years, with the rapid development of artificial intelligence techniques, researchers have tried to break through traditional methods and seek innovative examination and treatment schemes.

Preoperative Lauren classification has an important influence on the choice of treatment schemes. The infiltration of diffuse type GC often exceeds several centimeters of the boundary. Therefore, the resection scope of diffuse type GC is relatively large, and the margin of incision is about 8–10 cm away from the edge of the tumor [22]. On the other hand, diffuse type GC usually has a worse prognosis compared with intestinal type [23]. To this extent, it is particularly important to predict the Lauren classification accurately and preoperatively. In order to facilitate the study, the Lauren mixed type GC patients were removed as the clinical appearance and survival outcomes of mixed type are similar to diffuse type [2].

In this study, we extracted both tumor-based and peripheral ring-based radiomic features based on single CT image slices that were related to the Lauren classification. Research has suggested that 2D CT annotations might be a preferred choice in GC radiomics studies than 3D because 3D annotations might bring more noise [24]. Thus, our study was not limited by the use of single CT image slice. The LASSO method proved that 3 features were independent predictors. Then, instead of single factor analysis, we built and verified a radiomic nomogram for GC patients based on the combined radiomic signature representing tumors and the tumor surroundings in combination with clinical information including age, CT_T_, and CT_N_. Compared with 5 predictive models (combined radiomic signature, tumor-based model, peripheral ring-based model, clinical model 1, and clinical model 2), the radiomic nomogram achieved the highest predictive AUC, accuracy, and sensitivity. The accuracy (67.3%) was higher than the diagnostic accuracy of preoperative gastroscopic biopsy of the dataset in this study (65.7%), and also higher than the diagnostic accuracy of preoperative gastroscopic biopsy (64.7%) in Qiu et al.’s study [21].

Taking a step forward, the efficacy of our radiomic nomogram showed improvements compared to other noninvasive studies on the Lauren classification. Liu et al. [5] reviewed preoperative contrast-enhanced CT images and postoperative histopathological features of 107 GC patients, and they used parameters derived from portal venous CT images to distinguish diffuse type GC from others with an AUC of 0.655–0.674, less than the AUCs of our radiomic nomogram (0.758) on 539 cases. Ma et al. [4] analyzed DCE-MRI of 32 GC patients and found that the diffuse type showed a higher Ve and Ktrans than the intestinal type. However, short of study cases and model construction, it was not capable to be applied. Up till now, the radiomic nomogram we constructed is the most promising noninvasive prediction tool for the individualized Lauren classification in clinical trials.

As to the intratumoral and peritumoral analysis, the peripheral ring-based model demonstrated a general performance and failed to help promote the predictive ability of the combined radiomic signature significantly. Thus, peritumoral radiomics analysis may be inapplicable for predicting the Lauren classification. Concerning the clinical characteristics, the Lauren classification was significantly associated with age, CT_T_, and CT_N_ among the 539 cases in univariate analysis even after multiple comparison corrections. By interpretation from the radiomic nomogram, diffuse type GC was more likely to be associated with younger age, more advanced CT_T_, and more advanced CT_N_. These findings are still consistent with the previous studies [2, 25].

There are also some limitations in this study. First, this is a retrospective study and the CT images were obtained from two types of CT scanners, which may cause some bias and interference. Second, only venous phase CT images were used as it is difficult to identify tumor margins exactly on unenhanced or arterial phase images though they may contain some useful information. Finally, CT examination showed ionizing radiation, although the radiation dose in this study cohort was within the safe range for human body. With the acceleration of MRI and the application of motion artifact suppression technique, MRI without ionizing radiation may be a better choice.

## Conclusions

In conclusion, a radiomic nomogram was built by merging radiomic features of tumor and peritumor, age, CT_T_, and CT_N_ for the prediction of Lauren classification in GC preoperatively. The nomogram showed good performance in differentiating Lauren diffuse type from intestinal type in clinical treatment strategy management.

## Supplementary Information

**Additional file 1:** **Supplemental Method 1.** Computed tomography protocols and acquisition procedure. **Supplemental Method 2.** Filters in radiomic feature extraction. **Supplemental Method 3.** R packages in statistical analysis. **Supplemental Method 4.** Features in combined radiomic signature. **Supplemental Method 5.** Formulas for different predictive models. **Supplemental Figure 1.** Patient recruitment diagram. **Supplemental Table 1.** Multiple comparison corrections to adjust univariate *p* values for four clinical characteristics. **Supplemental Table 2.** Performances of the combined radiomic signatures based on 10-time dataset random allocations.

## Data Availability

The data and material are available through the corresponding author.

## Abbreviations

GC: Gastric cancer
CT: Computed tomography
AUC: Area under receiver operating characteristic curve
CI: Confidence interval
DCE-MRI: Dynamic contrast-enhanced magnetic resonance imaging
ROI: Region of interest
UICC: The Union for International Cancer Control
AJCC: American Joint Committee on Cancer
HE: Hematoxylin-eosin
ICC: Intraclass correlation coefficient
LASSO: Least absolute shrinkage and selection operator
