# Supplementary material for: Intratumoral and peritumoral radiomics analysis for preoperative Lauren classification in gastric cancer
Source: Cancer Imaging. 2020 Nov 23;20:83. doi: 10.1186/s40644-020-00358-3 (PMC7684959; doi:10.1186/s40644-020-00358-3)
Supplement: Supplementary file 1 — Additional file 1: Supplemental Method 1. Computed tomography protocols and acquisition procedure. Supplemental Method 2. Filters in radiomic feature extraction. Supplemental Method 3. R packages in statistical analysis. Supplemental Method 4. Features in combined radiomic signature. Supplemental Method 5. Formulas for different predictive models. Supplemental Figure 1. Patient recruitment diagram. Supplemental Table 1. Multiple comparison corrections to adjust univariate p values for four clinical characteristics. Supplemental Table 2. Performances of the combined radiomic signatures based on 10-time dataset random allocations. [file 40644_2020_358_MOESM1_ESM.docx]

**Additional file 1**

**Supplemental Method 1. Computed tomography protocols and acquisition procedure.**

Computed tomography (CT) images were acquired on 64-layer spiral CT (SOMATON sensation64, SIEMENS Healthcare, Germany) or 256-layer spiral CT (Brilliance iCT, ROYAL PHILIPS, Netherlands). The scanning parameters were: tube voltage, 120 kVp; tube current, 220~250 mA; detector collimation, 128×0.625 mm or 32×0.6 mm; reconstruction image thickness, 5 mm.

Patients fasting for 8 hours were screened on the empty stomach and intramuscularly administered with 20 mg hydrochloric acid anisodamine (654-2) in the first 10 minutes. Patients were asked to drink 800~1000 mL small mouth warm water to fill the stomach with pat on the back to reduce the gas volume. Then, an intravenous nonionic contrast medium iodine sea alcohol (iodine concentration of 270 mg / 100 mL) was injected with a volume of 1.5 mL/kg and an injection rate of 3.0 mL/s by the elbow. After injection of contrast medium for 35 s, arterial phase images were acquired and venous phase images were acquired after 70 s. The scanning range was from the top of diaphragm to the lowest margin of the liver or gastric body.

**Supplemental Method 2.** **Filters in radiomic feature extraction.**

Derived images were customized by wavelet, square, logarithm, exponential, gradient, and local binary pattern 2D filters. Detailed definitions are as follows.

Wavelet filtering in this study yielded 4 decompositions of the original image by applying either a High (H) or a Low (L) pass filter in either of the two dimensions, including HH, HL, LH, and LL. Square operations took the square of the image intensities and linearly scaled them back to the original range. Logarithm operations took the logarithm of the absolute intensity plus 1 and also scaled the values to the original range. Exponential operations got the exponential values of the absolute intensity of original image. Gradient filters returned the magnitude of the local gradient. Local binary pattern 2D filters computed the local binary pattern in a by-slice operation.

**Supplemental Method 3. R packages in statistical analysis.**

Intraclass correlation coefficients were calculated based on ‘psych’ package (version 1.7.8). The least absolute shrinkage and selection operator regression was carried out by ‘glmnet’ package (version 2.0-13). The multivariate logistic regression was performed using the ‘rms’ package (version 5.1-2). The receiver operating characteristic curves were conducted by ‘pROC’ package (version 1.10.0). Integrated discrimination improvement was measured by ‘PredictABEL’ package.

**Supplemental Method 4. Features in combined radiomic signature.**

1. *original_firstorder_Maximum_tumor* describes the maximum gray level intensity within the tumor-based region of interest (ROI) on the original image.
2. *wavelet.LL_firstorder_Maximum_tumor* is also a statistic describing the maximum gray level intensity within the tumor-based ROI, but on the derived image from LL wavelet filter.
3. *original_shape_Maximum2DDiameterSlice_peripheralring* quantifies the shape feature of the peripheral ring-based ROI on the original image. Maximum 2D diameter (Slice) is defined as the largest pairwise Euclidean distance between tumor surface mesh vertices in the axial plane.

**Supplemental Method 5. Formulas for different predictive models.**

$$Combined radiomic signature=0.2690\times original\_firstorder\_Maximum\_tumor+0.2936\times wavelet.LL\_firstorder\_Maximum\_tumor+0.5186\times original\_shape\_Maximum2DDiameterSlice\_peripheralring+0.1931$$

$$Tumor\_based model=0.5116\times original\_shape\_Maximum2DDiameterSlice\_tumor+0.2656\times original\_firstorder\_Maximum\_tumor+0.2960\times wavelet.LL\_firstorder\_Maximum\_tumor+0.1919$$

$$Peripheral\_ring\_based model=original\_shape\_Maximum2DDiameterSlice\_peripheralring$$

$$Radiomic nomogram=1.0578\times combined radiomic signature-0.0400\times age+0.2104\times IF\left( clinical T sgate=3 \right)+0.2414\times IF\left( clinical T stage=4 \right)-0.6418\times IF\left( clinical N stage=1 \right)-0.3009\times IF\left( clinical N stage=2 \right)+0.3442\times IF\left( clinical N stage=3 \right)+2.7062$$

**Supplemental Figure 1. Patient recruitment diagram.**


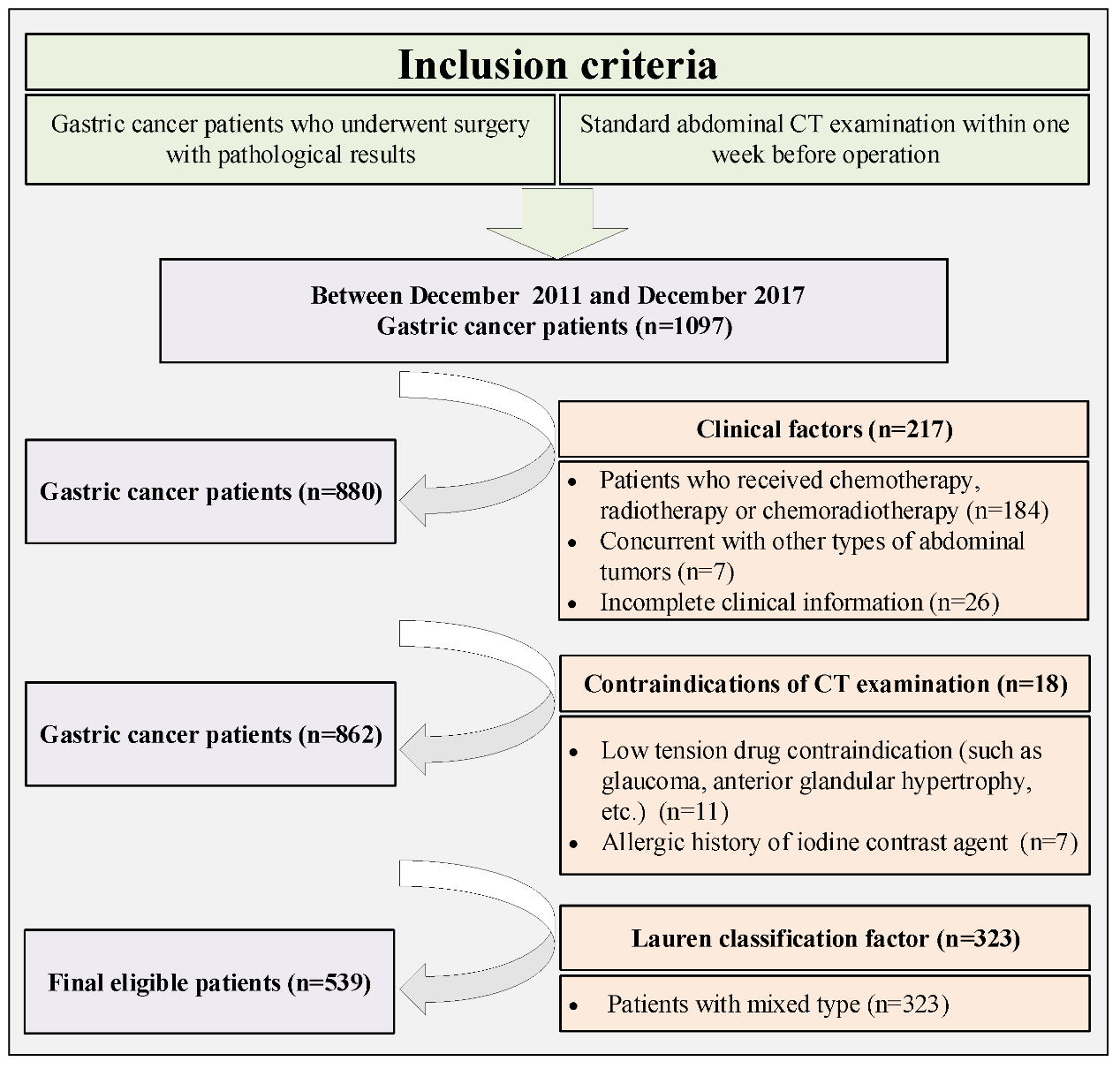


**Supplemental Table 1. Multiple comparison corrections to adjust univariate *p* values for four clinical characteristics.**

| **Clinical characteristics** | ***p* values** | **Adjusted *p* values** | |
| --- | --- | --- | --- |
|  |  | **Bonferroni correction** | **BH correction** |
| Age | 0.0042* | 0.0168* | 0.0122* |
| Sex | 0.0702 | 0.2808 | 0.0702 |
| CT T stage | 0.0247* | 0.0988 | 0.0329* |
| CT N stage | 0.0061* | 0.0244* | 0.0122* |

NOTE. Adjusted *p* values were calculated by Bonferroni correction and BH correction with four tests, respectively. *represents a statistically significant level. Abbreviations: CT, computed tomography; BH correction, Benjamini & Hochberg correction.

**Supplemental Table 2. Performances of the combined radiomic signatures based on 10-time dataset random allocations.**

| **Experiments** | **Total selected features**  **(tumor ROI + peripheral ring)** | **Combined radiomic signature** | |
| --- | --- | --- | --- |
|  |  | **Training AUC** | **Validation AUC** |
| 1 | 4 (3+1) | 0.715 | 0.711 |
| 2 | 2 (2+0) | 0.715 | 0.699 |
| 3 | 5 (2+3) | 0.705 | 0.754 |
| 4 | 2 (2+0) | 0.712 | 0.710 |
| 5 | 3 (3+0) | 0.714 | 0.706 |
| 6 | 3 (2+1) | 0.718 | 0.694 |
| 7 | 3 (2+1) | 0.715 | 0.708 |
| 8 | 6 (4+2) | 0.734 | 0.678 |
| 9 | 2 (1+1) | 0.705 | 0.726 |
| 10 | 2 (2+0) | 0.716 | 0.701 |
| Average | / | 0.715 | 0.709 |
| Ours | 3 (2+1) | 0.715 | 0.714 |
| Delta | / | 0 | -0.005 |

NOTE. The average AUCs of the 10 experiments were 0.715 and 0.709 in both cohorts, very close to the performance of the combined radiomic signature proposed in this study (0.715 and 0.714), indicating that our methods based on the dataset in this study were stable. Abbreviations: ROI, region of interest; AUC, area under the curve.
